# Modern technology complements classic technique: 3D-printed inter-locking cutting guides for frontal sinus osteoma resection and reconstruction with split-calvarial bone graft

**DOI:** 10.1080/23320885.2026.2676455

**Published:** 2026-06-03

**Authors:** Caleb W. Brown, Taylor A. Martin, Diego E. Razura, Abbey L. G. Johnson, Neil B. Horsley, Thomas M. Soike, Jeremy M. Powers

**Affiliations:** a Quillen College of Medicine, East Tennessee State University, Johnson City, TN, USA; b Department of Internal Medicine, Boston Medical Center – Brighton, Boston, MA, USA; c Department of Otolaryngology - Head and Neck Surgery, Mayo Clinic College of Medicine and Science, Phoenix, AZ, USA; d Department of Surgery, Quillen College of Medicine, East Tennessee State University, Johnson City, TN, USA; e Department of Pathology, Quillen College of Medicine, East Tennessee State University, Johnson City, TN, USA; f Department of Surgery, Division of Plastic and Reconstructive Surgery, Quillen College of Medicine, East Tennessee State University, Johnson City, TN, USA

**Keywords:** Calvarial graft, frontal sinus, frontal sinus reconstruction, osteoma, virtual surgical planning

## Abstract

**Introduction:**

Frontal sinus osteomas are rare benign tumors that may cause functional or cosmetic concerns. Virtual surgical planning (VSP) offers promising adjuncts to classic craniofacial techniques to improve surgical precision and minimize long-term morbidity.

**Patient:**

A previously healthy 15-year-old male presented with a two-year history of headaches and a slowly enlarging mid-forehead mass. Imaging revealed a 3 × 2 cm frontal sinus osteoma with mucocele formation and erosion of both the anterior and posterior tables, with early mass effect on the frontal lobes. VSP was used to design bi-frontal craniotomy and split-calvarial bone graft cutting guides. A combined approach by plastic surgery and neurosurgery was performed using resorbable plating for reconstruction.

**Results:**

The operation proceeded without complication. The osteoma was excised, cranialization of the frontal sinus was completed, and the frontal bone was reconstructed using a split-calvarial bone graft secured with resorbable fixation. The patient recovered uneventfully and was discharged on postoperative day four. At six-month, one-year and two-year follow-up, he remained asymptomatic, with six-month imaging showing stable reconstruction and no tumor recurrence. Pathology confirmed a mixed-type osteoma.

**Conclusion:**

This case illustrates the successful application of VSP, split-calvarial grafting, and resorbable plating for the resection and reconstruction of a frontal sinus osteoma. The use of patient-specific guides allowed for precise resection and autologous reconstruction. This technique may serve as a valuable model for similar cases in craniofacial surgery.

## Introduction

Osteomas are the most common primary tumors of craniofacial osseous tissue. These benign tumors are most frequently reported in the mandible, maxilla, cranial vault, external auditory canal, and orbit. However, they also occur in the paranasal sinuses with an estimated prevalence of 3% [1]. Most paranasal osteomas are located in the frontal sinuses, followed by the ethmoid and maxillary sinuses, with rare occurrences in the sphenoid sinuses [1,2]. These slow-growing masses may cause discomfort, mucocele, and carry the potential for erosion into adjacent calvarial structures [3]. While often asymptomatic, surgical intervention becomes necessary when symptoms such as headaches, dizziness, diplopia, proptosis, sinusitis, facial deformity, or concurrent infection occur [4]. Effective surgical planning requires consideration of factors such as tumor size, growth rate, erosion into surrounding structures, and predominant symptoms. It is crucial to prioritize reducing harm to adjacent structures and optimizing aesthetic outcomes when planning a surgical approach.

Virtual surgical planning (VSP) is an innovative and viable pre-operative planning option in the complex surgical management of paranasal sinus osteomas. VSP allows physicians to engage with virtual models customized to individual patients, facilitate the simulation of surgical procedures, and enhance the planning process [5]. Surgeons have access to various VSP elements, such as three-dimensional virtual planning, stereolithographic models, intraoperative cutting guides, intraoperative dental splints, and patient-specific implants [6]. The application of VSP has demonstrated increasing utility in pediatric craniofacial surgery, showing a 5.3% increase in use from 2009 to 2018 [6]. This increase in VSP for these specific indications is likely due to increased availability, improved precision of resections and reconstructions, reduction of operative time, reduced complication rates, and enhanced consistency of results [5].

In craniofacial reconstruction, the choice of graft material must account for long-term durability, integration, and compatibility with the surrounding anatomy. Split-calvarial autograft remains a widely utilized option due to its inherent biocompatibility and capacity to integrate seamlessly with native bone, reducing the risk of long-term implant-related complications associated with alloplastic materials. These properties make autologous calvarial bone an appealing reconstructive choice in pediatric and adolescent patients whether or not they have reached skeletal maturity.

We present a case report of a 15-year-old male diagnosed with a frontal sinus osteoma whose surgical resection and reconstruction was facilitated by virtual surgical planning and 3D-printed cutting guides. Long term follow up has demonstrated durable reconstruction with excellent outcome.

## Patient

**Figure 1.**
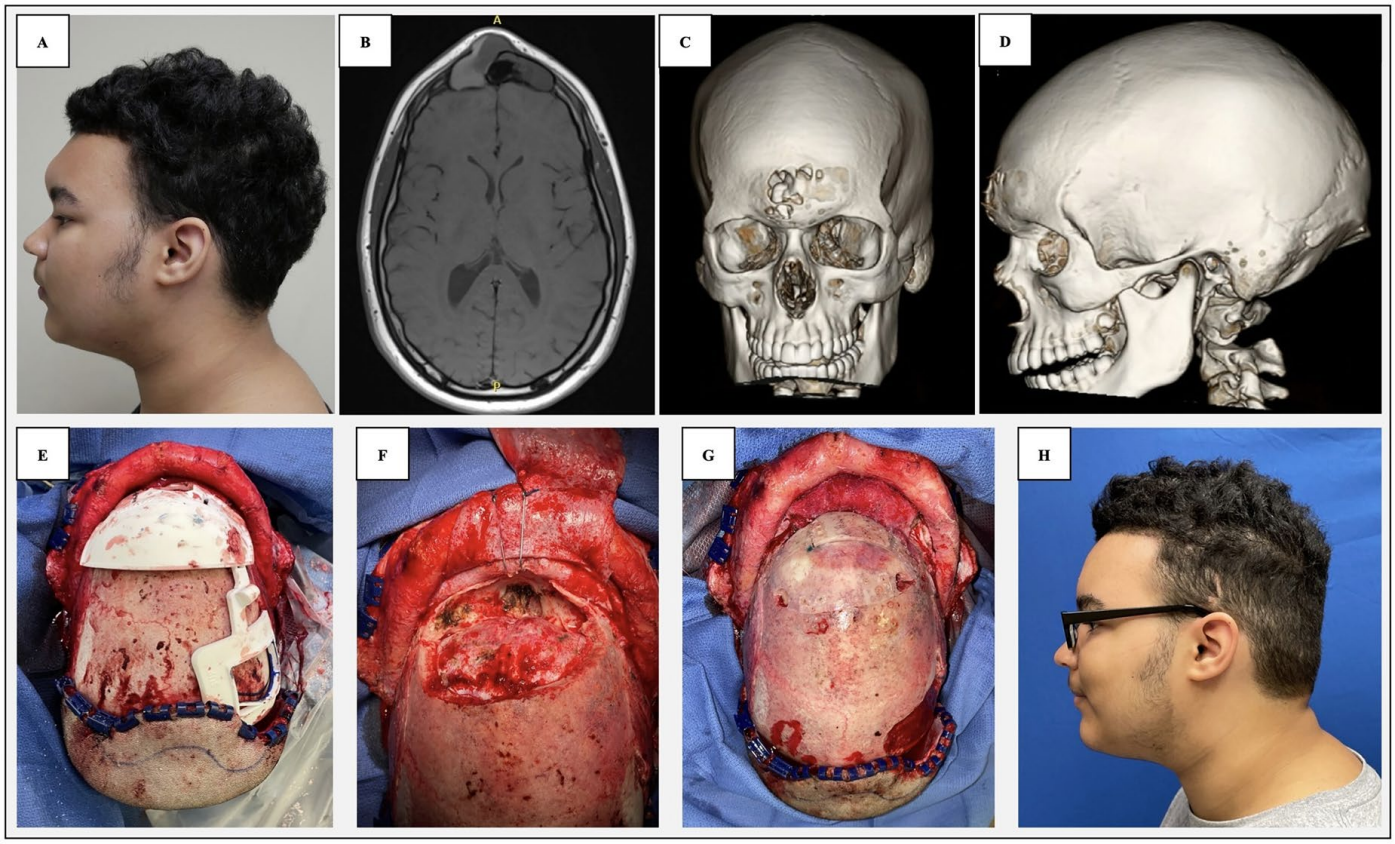
(A) Preoperative left profile of frontal sinus osteoma. (B) Preoperative magnetic resonance imaging with contrast, axial view. (C) Preoperative 3-dimensional computed tomography (CT) scan, frontal view. (D) Preoperative 3-dimensional CT scan, profile view. (E–G) Intraoperative frontal sinus reconstruction with split-calvarial bone graft and KLS Martin resorbable fixation system. (H) Postoperative left profile at 7-month follow-up.

A previously healthy 15-year-old male presented with a two-year history of headaches in the setting of a prominent 3 × 2 cm mass extending from the lower forehead to the glabella (Figure 1). The mass was firm to palpation inferiorly but noted to be soft in its superior aspect. Computed Tomography (CT) imaging (Figure 1) supported the diagnosis of osteoma centered in the frontal sinus with an associated mucocele eroding the outer and inner tables of the frontal bone. The lesion was noted to extend posteriorly, causing early mass effect on the frontal lobes. Due to the risk of eventual intracranial involvement, further erosion into the bony structures, and infection, the decision was made to proceed with surgical resection. Neurosurgical consultation was obtained with plans for a combined surgery. Additionally, the decision was made to utilize VSP with the goal of increasing the precision of resection and optimizing reconstruction outcomes with minimal permanent hardware.

Virtual surgical planning and guide production were performed in collaboration with KLS Martin Group (Tuttlingen, Germany). Raw DICOM (Digital Imaging and Communications in Medicine) data from a thin-slice CT scan of the head and facial bones were provided to a KLS Martin biomedical engineer. A virtual planning session was then conducted between the operative surgeon and engineer to guide the design of the cutting guides. Through this collaborative process, patient-specific guides were developed based on the planned resection and reconstruction. The finalized guides were fabricated using 3D printing and delivered to the institution for sterilization prior to surgery.

**Figure 2.**
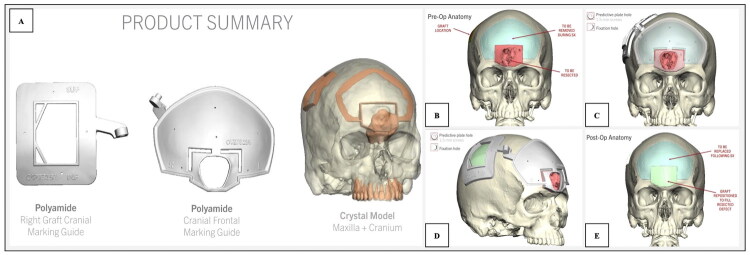
(A) Cutting guides for split-calvarial bone graft. (B) Virtual preoperative anatomy. (C,D) Predicted intraoperative placement of guides. (E) Anticipated postoperative anatomy.

First, a cutting guide was designed for bifrontal craniotomy. Markings were incorporated into the frontal cutting guide, featuring an outer border and an inner border (Figure 2). The outer border marked the bifrontal craniotomy cut for removal of sufficient frontal bone to widely access the anterior skull base. The inner border marked the necessary resection of outwardly deformed frontal bone, which was to be reconstructed with a split-calvarial bone graft. An interlocking cutting guide was created that facilitated split-calvarial bone graft harvest from the right parietal skull. Adequate thickness of bone graft from the non-dominant hemisphere was considered for safe graft harvest. The bony curvature of the parietal bone at the site of bone graft harvest was perfectly aligned with the desired curvature and contour of the frontal bone reconstruction by VSP engineers. The interlocking aspect of the cutting guides ensured that the location and shape of the split-calvarial bone graft would be an exact match to the inner border of the frontal cutting guide (Figure 2).

A multidisciplinary intraoperative approach was used with the presence of plastic surgery and neurosurgery throughout the case. Coronal approach *via* sinusoidal incision was performed, with separate dissection of a pericranial flap to the level of the frontal bar. Bifrontal craniotomy was performed with removal of the frontal bone flap to access the anterior skull base. The bony tumor at the frontal sinus and anterior skull base was mechanically resected using a series of drills and cutting burrs. A combined endoscopic endonasal approach was not utilized in this case due to the size and density of the bony lesion.

Any remaining frontal sinus mucosa was obliterated, and the rest of the posterior table of the frontal sinus was removed to complete a cranialization. The pericranial flap was placed into the skull base to separate the paranasal sinus from the intracranial space. A resorbable mesh was contoured to the skull base defect and placed over the pericranial flap. The flap was then reflected over the resorbable plate, with the remaining flap tissue folded onto itself to eliminate any residual dead space anterior to the frontal lobes.

The bifrontal craniotomy bone flap was then replaced *in situ* and fixated with a KLS Martin resorbable system (SonicWeld Rx^®^, KLS Martin Group, Tuttlingen, Germany). The split-calvarial bone graft was harvested from the right parietal bone according to the markings from the interlocking cutting guide. DBX bone putty^®^ (MTF Biologics, Edison, NJ) was used to fill the graft harvest site and burr holes to prevent contour irregularities. The split-calvarial bone graft was used to reconstruct the central bony defect in the frontal bone and was secured with additional resorbable plates. Following irrigation and hemostasis, Jackson-Pratt (JP) drains were placed under the anterior and posterior scalp flaps, and the scalp incision was closed in layers with absorbable suture.

## Results

**Figure 3.**
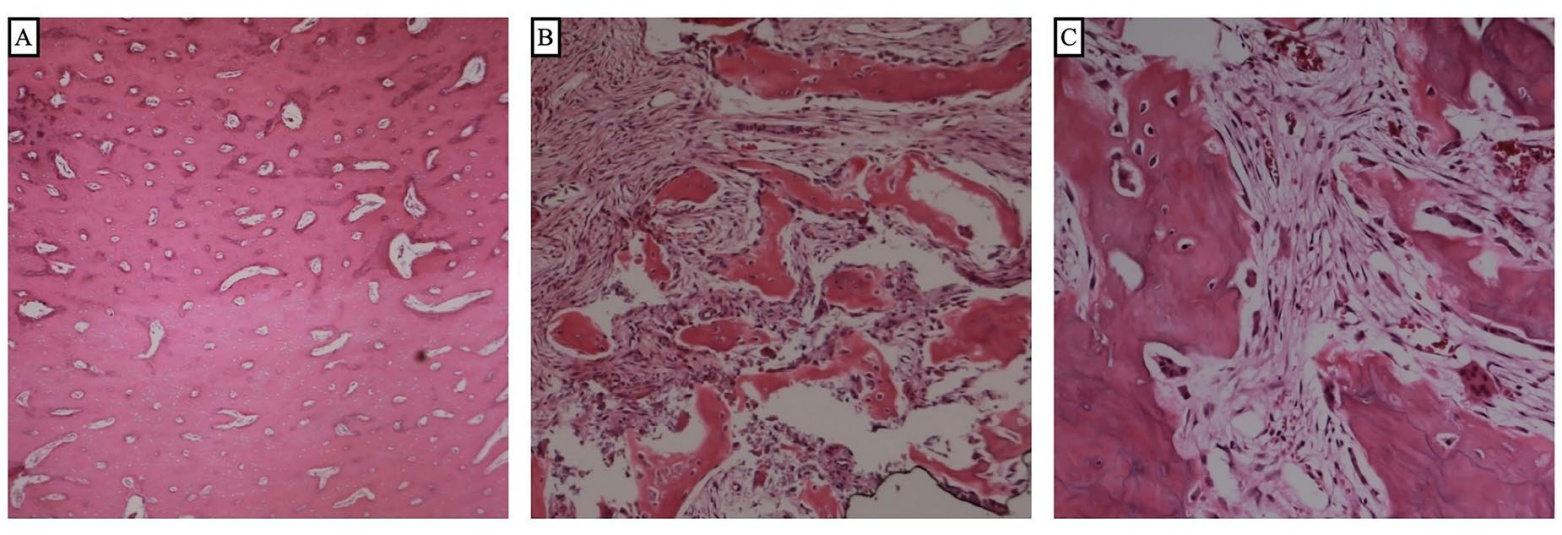
Representative hematoxylin and eosin (H&E)–stained histologic sections show interwoven compact dense bone (A, 40×) and cancellous bone (B, 100×) with trabeculae of bone and marrow. Minor components of the tissue mimic a more aggressive appearing osteoblastoma (C, 200×), with plump osteoblasts rimming woven bone. These osteoblastoma-type areas are encapsulated by dense woven bone. No significant cytologic atypia is noted, and the mitotic rate is low.

The patient was admitted to the pediatric intensive care unit (PICU) post-operatively and discharged home on post-operative day 4 following an unremarkable post-operative course. Pathology confirmed that the excised lesion was a mixed-type osteoma (Figure 3). CT imaging at 6 months post-resection confirmed no tumor recurrence with routine bony healing. The patient has remained asymptomatic and without post-operative complications.

## Discussion

Frontal sinus osteomas are benign, slow-growing tumors that may cause symptoms through mass effect, obstruction of sinus drainage, or cosmetic deformity. Common presentations include headache, facial pain, recurrent sinusitis, mucocele formation, and orbital findings such as proptosis [7]. Surgical resection is indicated for symptomatic lesions, rapidly enlarging tumors, or those with intracranial or intraorbital extension, with primary goals of complete tumor removal, preservation of adjacent critical structures, and restoration of the frontal contour [8].

Multiple imaging modalities are available for evaluating frontal bone and sinus pathology, each offering distinct diagnostic advantages. CT and magnetic resonance imaging (MRI) remain the primary tools for sinonasal assessment, with CT providing detailed characterization of bony anatomy essential for operative planning, and MRI offering superior soft tissue resolution for detecting intracranial or intraorbital extension and distinguishing masses from retained secretions [9]. Additionally, ultrasonography has emerged as a useful adjunct imaging modality, particularly in outpatient settings and resource-limited environments. Sinus sonography demonstrates high sensitivity and specificity in identifying sinus pathology, can guide triage for advanced imaging, and has potential utility in pediatric and pregnant patients where radiation exposure is a concern [10]. Together, these modalities enable a tailored and comprehensive approach for evaluating frontal sinus lesions and planning surgical intervention.

The choice of approach is determined by tumor size, location, and extension. In a systematic review of 477 surgically treated cases, transnasal endoscopic resection was most common (44.9%), followed by open osteoplastic flap (36.9%) and combined endoscopic-assisted techniques (18.2%) [7]. Endoscopic techniques are favored for small, medially located lesions due to their minimally invasive nature, direct visualization, and favorable cosmetic outcomes [8]. However, large, laterally located, or extensively invasive tumors are more safely addressed *via* open approaches, such as coronal craniotomy, which provide wide exposure and facilitate complete resection. Reconstruction of the anterior frontal sinus wall after open resection has been achieved with titanium mesh [11], porous titanium [12], porous polyethylene [13], polyetheretherketone (PEEK) [14], hydroxyapatite [15], or autologous split-calvarial bone grafts as in this case. The latter remains a time-tested method, harvested from the outer parietal table using an oscillating saw and osteotomes, then manually contoured intraoperatively. While durable, this technique is technically demanding and often requires iterative adjustments to achieve optimal fit and contour.

VSP has emerged as a valuable adjunct in craniofacial surgery, allowing translation of precise preoperative designs into the operating room through patient-specific instrumentation. The process begins with standardized imaging, most often high-resolution CT or cone-beam CT, with optional integration of 3D surface scans, photographs, or MRI [16]. Segmentation techniques create discrete anatomical models, which are registered and aligned using algorithms to produce an accurate 3D digital workspace. The surgeon and biomedical engineer then collaborate to perform virtual osteotomies and design surgical guides, which are manufactured *via* 3D printing in biocompatible materials. These guides enable highly accurate intraoperative execution of the planned osteotomies and allow preoperative contouring of reconstruction materials [16].

Over the past two decades, VSP has evolved from basic templates to an integrated platform for tumor resection, trauma reconstruction, orthognathic surgery, and skull base approaches. Studies consistently demonstrate smaller deviations between planned and actual outcomes with VSP compared to conventional methods, with mean linear discrepancies as low as 0.04–0.25 mm versus 0.29–1.33 mm for non-VSP [17]. Although meta-analyses have not always shown statistically significant differences across all outcomes, trends toward reduced operative time, fewer intraoperative adjustments, and improved graft-recipient congruence have been observed [18]. The use of VSP has been reported to decrease operative time by 1.3 h in cranial vault remodeling for craniosynostosis (3.7 ± 1.1 vs. 5.0 ± 1.1 h; *p* < 0.001) and to reduce mean operative time in microvascular head and neck reconstruction (507.38 vs. 561.75 min; *p* = 0.042), further demonstrating the efficiency advantages associated with virtual surgical planning [19,20].

Although virtual surgical planning (VSP) has demonstrated workflow advantages, its cost-effectiveness remains variable. Most economic evaluations suggest that VSP can be cost-neutral or even cost-saving compared to freehand surgery, largely through reductions in operative time and hospital stay; however, cost benefits are most pronounced in centers with in-house planning capabilities, whereas outsourced workflows introduce added expense and logistical barriers that may limit broader adoption [21]. Meta-analytic data similarly reveal cost trends favoring VSP, though findings did not reach statistical significance, in part due to heterogeneity in study design and outcome reporting [18].

International generalizability also remains uncertain, as much of the existing literature originates from a limited number of high-resource countries, and cost analyses are underreported in many regions where access to digital planning infrastructure is constrained [5]. Recent literature further describes the evolving workflow of VSP, including in-house design and manufacturing approaches [22]. However, centers that may not yet have the clinical volume or infrastructure to support such systems can effectively utilize collaboration with industry-based biomedical engineering teams, as demonstrated in the present case. These limitations highlight the need for standardized, procedure-specific economic studies and expanded global datasets to better define the feasibility and scalability of VSP across diverse healthcare systems.

In frontal sinus surgery, VSP offers distinct advantages due to the region’s complex and variable anatomy, proximity to the anterior skull base, and risk to intracranial and orbital structures. By mapping the lesion and designing osteotomies directly over it, VSP allows tailored osteoplastic flap creation that optimizes exposure while minimizing unnecessary bone removal [23]. Inter-locking cutting guides extend these benefits by virtually matching donor and recipient sites. In this configuration, one guide shapes the defect while its counterpart shapes the donor graft—such as a split-calvarial segment—to fit precisely, eliminating intraoperative contouring and preserving curvature and thickness relationships planned in the virtual model.

In the present case, this strategy allowed the defect-site osteotomy and parietal split-calvarial harvest to be executed as complementary, perfectly matched cuts. The result was a reconstruction that required no intraoperative adjustment of the graft, preserved the integrity of the donor site, and maintained the native frontal curvature. By merging modern digital planning with established craniofacial techniques, we achieved an accurate, efficient, and durable repair.

A systematic literature review of surgical management of frontal sinus osteomas in young patients identified 35 relevant publications, of which 6 were selected for detailed analysis. Across these reports, surgical management strategies for frontal sinus osteomas varied widely, and no prior case was identified combining VSP, inter-locking cutting guides, and split-calvarial bone grafting. Kim and colleagues described a similar reconstructive approach without VSP [24], and variation in other published cases highlight the lack of a standardized reconstructive protocol for large anterior wall defects (Table 1) [13,25–29]. Additionally, while recent literature supports the benefits of VSP in the management of facial osteomas in adult populations and highlights its transformative impact on pediatric craniofacial surgery, reports describing its use in young patients with frontal sinus osteomas are limited [30,31].

**Table 1.** Summary of pediatric frontal sinus osteoma cases identified in the literature.

Paper	Age/sex	Diagnosis	Presentation	Location	Procedure	Outcome
Current Case Report, 2025	15/M	Osteoma with mucocele	Enlarging mass; Chronic headaches	Centered in the frontal sinus	Virtual surgical planning (VSP) with KLS Martin resorbable plate for reconstruction	No complications
Hosseini et al. 2016	14/M	Osteoma and fibrous dysplasia	Enlarging frontal sinus bulge	Frontal sinus, extending to the left orbit and ethmoid sinus	En bloc resection with frontal sinus reconstruction using Porex (MEDPOR)	No complications; CT scan 4 years post-operation revealed appropriate reconstruction
Jack et al. 2009	16/M	Osteoma	Orbital emphysema	Right frontal sinus	Anterior orbitotomy with reconstruction using nylon foil	No complications; no post-operative episodes of orbital emphysema
Shady et al. 1994	17/F	Osteoma with mucocele	Acute and chronic sinusitis; recurrent vomiting, diplopia, and blurred vision	Left frontal sinus	Left frontal osteoplastic craniotomy; osteotomy of anterior wall of left frontal sinus; reconstruction with microplate	No complications; no post-operative episodes of sinusitis
Innis et al. 2005	16/M	Osteoma	Persistent left frontal pain	Left frontal sinus	Image-guided osteoplastic frontal sinusotomy (OFS) technique using LandmarX Evolution	No complications
Kim et al. 2000	13/F	Osteoma	Left periorbital pain and reported diplopia on upgaze; mild proptosis of left orbit	Left frontal sinus, extending into the left orbit	Superior craniotomy approach with bioabsorbable fixation system used for repair	No complications; decreased proptosis with residual left hypoglobus
Strek et al. 2007	15/M	Osteoma	Frontal headache	Right frontal sinus	External osteoplastic procedure	No complications; Symptom improvement

This table highlights previously reported pediatric frontal sinus osteoma cases, including presenting symptoms, tumor location, operative approaches, and outcomes. Although various surgical techniques have been utilized for osteoma removal and sinus reconstruction in this population, none of the prior cases incorporated virtual surgical planning or patient-specific, inter-locking 3D-printed cutting guides. The present case represents the first reported use of these advanced digital planning technologies in pediatric frontal sinus osteoma management, underscoring the novelty and potential clinical utility of this approach.

This report is limited by the inherent constraints of a single-case experience, which preclude generalization. While the technical benefits of VSP and inter-locking guides were clear in this case, further comparative studies are warranted to quantify improvements in operative efficiency, graft fit, and long-term outcomes relative to traditional methods.

## Conclusion

This case demonstrates how modern digital tools can enhance established craniofacial surgical principles. By integrating VSP with inter-locking cutting guides, we achieved a precise, efficient, and anatomically faithful reconstruction of a large frontal sinus defect using a split-calvarial bone graft. This approach minimized intraoperative adjustments, preserved donor site integrity, and maintained the natural curvature of the forehead, addressing the unique anatomical challenges of the frontal sinus while leveraging the durability of classic techniques. Furthermore, we are confident that the expanding capabilities of VSP and patient-specific 3D-printed guides could prove beneficial in other reconstructive arenas, including complex craniofacial trauma, warranting further investigation.

## Data Availability

The data that support the findings of this case report are not publicly available due to privacy restrictions.
